# Large-scale transcriptome comparison of sunflower genes responsive to *Verticillium dahliae*

**DOI:** 10.1186/s12864-016-3386-7

**Published:** 2017-01-06

**Authors:** Shuchun Guo, Yongchun Zuo, Yanfang Zhang, Chengyan Wu, Wenxia Su, Wen Jin, Haifeng Yu, Yulin An, Qianzhong Li

**Affiliations:** 1 Laboratory of Theoretical Biophysics, School of Physical Science and Technology, Inner Mongolia University, Hohhot, 010021 China; 2 Inner Mongolia Academy of Agricultural & Animal Husbandry Sciences, Hohhot, 010031 China; 3 The Key Laboratory of Mammalian Reproductive Biology and Biotechnology of the Ministry of Education, College of life sciences, Inner Mongolia University, Hohhot, 010021 China

**Keywords:** *Helianthus annuus L*, *Verticillium dahliae*, RNA-Seq, Differentially expressed genes (DEGs), Defense responses genes

## Abstract

**Background:**

Sunflower Verticillium wilt (SVW) is a vascular disease caused by root infection with *Verticillium dahliae (V. dahlia)*. It is a serious threat to the yield and quality of sunflower. However, chemical and agronomic measures for controlling this disease are not effective. The selection of more resistant genotypes is a desirable strategy to reduce contamination. A deeper knowledge of the molecular mechanisms and genetic basis underlying sunflower Verticillium wilt is necessary to accelerate breeding progress.

**Results:**

An RNA-Seq approach was used to perform global transcriptome profiling on the roots of resistant (S18) and susceptible (P77) sunflower genotypes infected with *V. dahlia*. Different pairwise transcriptome comparisons were examined over a time course (6, 12 and 24 h, and 2, 3, 5 and 10 d post inoculation). In RD, SD and D datasets, 1231 genes were associated with SVW resistance in a genotype-common transcriptional pattern. Moreover, 759 and 511 genes were directly related to SVW resistance in the resistant and susceptible genotypes, respectively, in a genotype-specific transcriptional pattern. Most of the genes were demonstrated to participate in plant defense responses; these genes included peroxidase (POD), glutathione peroxidase, aquaporin PIP, chitinase, L-ascorbate oxidase, and LRR receptors. For the up-regulated genotype-specific differentially expressed genes (DEGs) in the resistant genotype, higher average fold-changes were observed in the resistant genotype compared to those in the susceptible genotype. An inverse effect was observed in the down-regulated genotype-specific DEGs in the resistant genotype. KEGG analyses showed that 98, 112 and 52 genes were classified into plant hormone signal transduction, plant-pathogen interaction and flavonoid biosynthesis categories, respectively. Many of these genes, such as CNGC, RBOH, FLS2, JAZ, MYC2 NPR1 and TGA, regulate crucial points in defense-related pathway and may contribute to *V. dahliae* resistance in sunflower.

**Conclusions:**

The transcriptome profiling results provided a clearer understanding of the transcripts associated with the crosstalk between sunflower and *V. dahliae*. The results identified several differentially expressed unigenes involved in the hyper sensitive response (HR) and the salicylic acid (SA)/jasmonic acid (JA)-mediated signal transduction pathway for resistance against *V. dahliae*. These results are useful for screening resistant sunflower genotypes.

**Electronic supplementary material:**

The online version of this article (doi:10.1186/s12864-016-3386-7) contains supplementary material, which is available to authorized users.

## Background

**Fig. 1:**
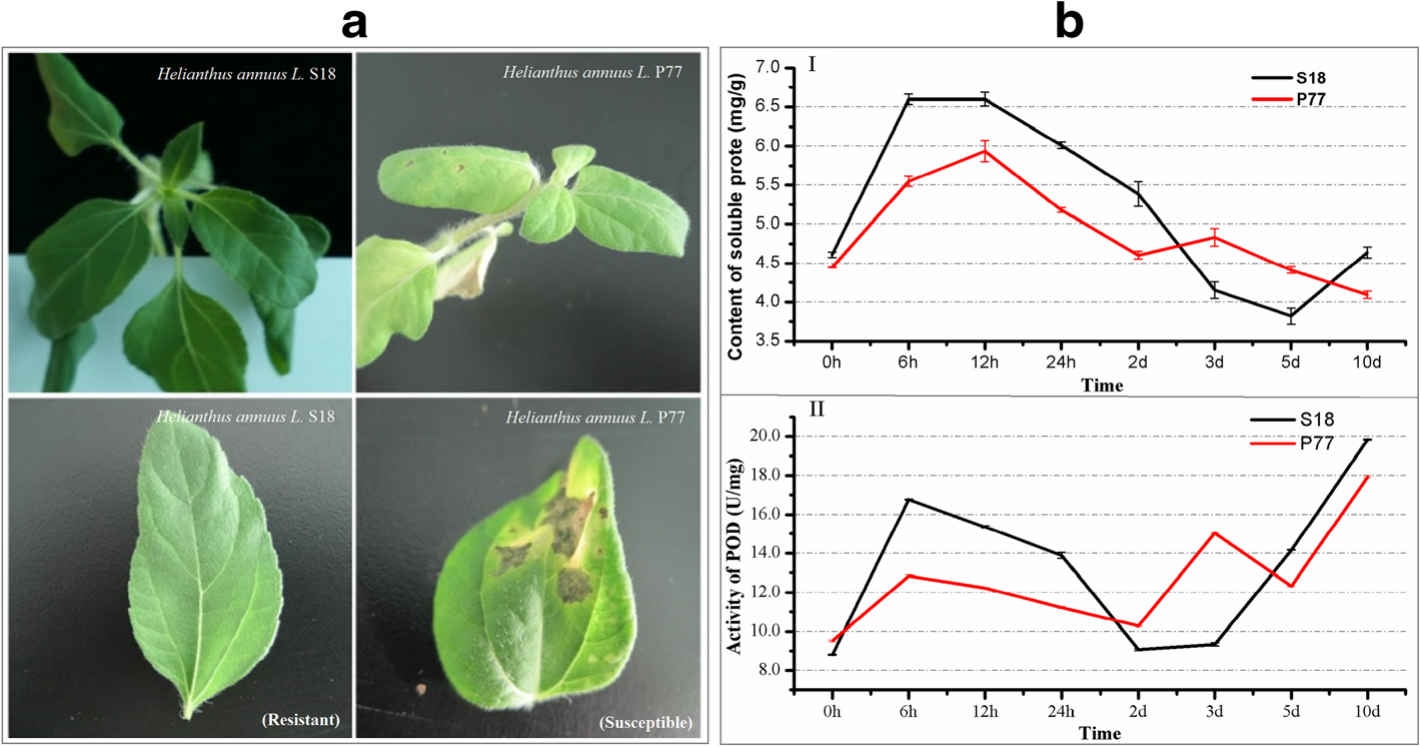
Symptoms and dynamics of the main physiological indexes in inoculated resistant genotype (S18) and susceptible genotype (P77). **a** Symptoms of the resistant and susceptible genotypes 14 d after *V. dahliae* inoculation. **b** Dynamics of the main physiological indexes in the inoculated resistant and susceptible genotypes at different time points after inoculation. I Soluble protein, II POD

Sunflower (*Helianthus annuus L.*) is an important oil crop and ornamental plant that is also considered an efficient source of biodiesel [1, 2]. Because sunflowers have the characteristics of drought resistance and strong salinity tolerance, these plants have been widely planted throughout the world. However, with the gradual expansion of the sunflower planting area, diseases also occur. In particular, the worldwide spreads of Sunflower Verticillium wilt (SVW) has become the main disease currently affecting sunflower production. SVW, a serious soil-borne vascular disease is caused by *Verticillium dahliae* [3], which is harmful to more than 200 plant species worldwide [4, 5]. This disease causes sunflower leaves to become yellow or withered, followed by the eventual death of seriously infected plants (Fig. 1a).

In recent years, efforts had been made in discovering the molecular mechanisms underlying the interactions between *V. dahliae* and Arabidopsis, tomato, potato, and cotton plants, and several resistance-related genes and enzymes have been well characterized [6–10]. In the interaction between Arabidopsis thaliana and Verticillium Wilt, Pantelides reported enhanced resistance in etr1-1 [ethylene (ET) receptor mutant] plants, indicating a crucial role for ETR1 in defense against this pathogen, and quantitative polymerase chain reaction analysis suggested that the impaired perception of ET via ETR1 results in increased disease resistance [11]. In a previous study, Yao showed that NO may act as an upstream signaling molecule to trigger the deploymerization of cortical microtubules in Arabidopsis [12]. In 2014, a study showed that Ve chimeras in which the first thirty eLRRs of Ve1 were replaced with those of Ve2 retain the ability to induce HR, and the Ve1 gene of tomato confers resistance against race 1 strains of *V. dahliae* and V. albo-atrum [13, 14]. Song, Y et al. suggested an ancient origin of the Ve1 immune receptor in the plant kingdom [15]. In 2015, RNA-Seq analysis showed that disease defense genes were expressed at much higher levers in the tomatoes grown with potato or onion plants than in tomato plants grown alone [16]. Furthermore, some studies have speculated that a miR482-mediated silencing cascade is involved in the regulation of potato resistance against *V. dahliae* infection and in the counter defense action of plants in response to pathogen infection [17]. In studies on cotton responses to *V. dahliae*, Sun et al. used the RNA-Seq method to identify 44 differentially expressed genes involved in cotton defense responses [18]. Using the same method, a total of 3027 unigenes were determined as be homologous to known defense-related genes in other plants [19].

No fungicides are available to cure infected commercial sunflowers. Thus, resistance breeding is the most ideal strategy for controlling plant pathogenic fungus with great benefit of economy and environmental protection. In the present study, we used RNA-Seq to discuss and compare the transcriptome profile in the roots of resistant and susceptible sunflower genotypes under *V. dahliae* infection. Different pairwise transcriptome comparisons were made over a time course (6, 12 and 24 h, and 2, 3, 5 and 10 days post-inoculation). A comparison of the uninoculated resistant genotype with the uninoculated susceptible genotype showed a basal gene expression pattern. Genotype-common and genotype-specific transcriptional changes in response to *V. dahliae* inoculation were further identified. The potential roles for DEGs were researched, and the resistance mechanism of sunflower against *V. dahliae* was also discussed. These finding will contribute to a better understanding of the molecular interactions between sunflower and *V. dahlia* for resistance breeding and provide insight into the interactions between plants and pathogens.

## Results

### Study of the main physiological indexes in sunflower roots post-inoculation

Two sunflower genotypes, previously classified as resistant (S18) and susceptible (P77) to Sunflower Verticillium wilt (SVW) according to their field behavior, were used in this study (Fig. 1a). To investigate the process of SVW colonization, 3 indexes (Soluble protein, Peroxidase (POD), and Malondialdehyde (MDA)) were measured at 6, 12 and 24 h, and 2, 3, 5 and 10 days post inoculation for the two sunflower genotypes. After infection with *V. dahliae*, the content of soluble protein initially increases, but then decrease thereafter, and the contents peaked from 6 to 12 h when treatment was prolonged. The physiological index in the resistant genotype was significantly higher than that in the susceptible genotype at 6, 12, and 24 h and 2 d. However, in the resistant genotype, the physiological index was lower than that in the susceptible genotype at 3 and 5 d, and this reversed at 10 d (Fig. 1b). The POD activity in the resistant genotype was higher at 6, 12, and 24 h, and 2, 5 and 10 d compared with the susceptible genotype (Fig. 1b). For MDA measurement, the content was slightly higher in the susceptible genotype than in the resistant genotype at the four initial time points (Additional file 1: Figure S1). Based on the above discussion of the physiological indexes, we concluded that the resistant genotype performed better in resistance to *V. dahliae* infection. To further identify the resistance-related genes, a RNA-Seq experiment for all time points examined above was proposed in the following study.

### Illumina sequencing and de novo assembly

**Fig. 2:**
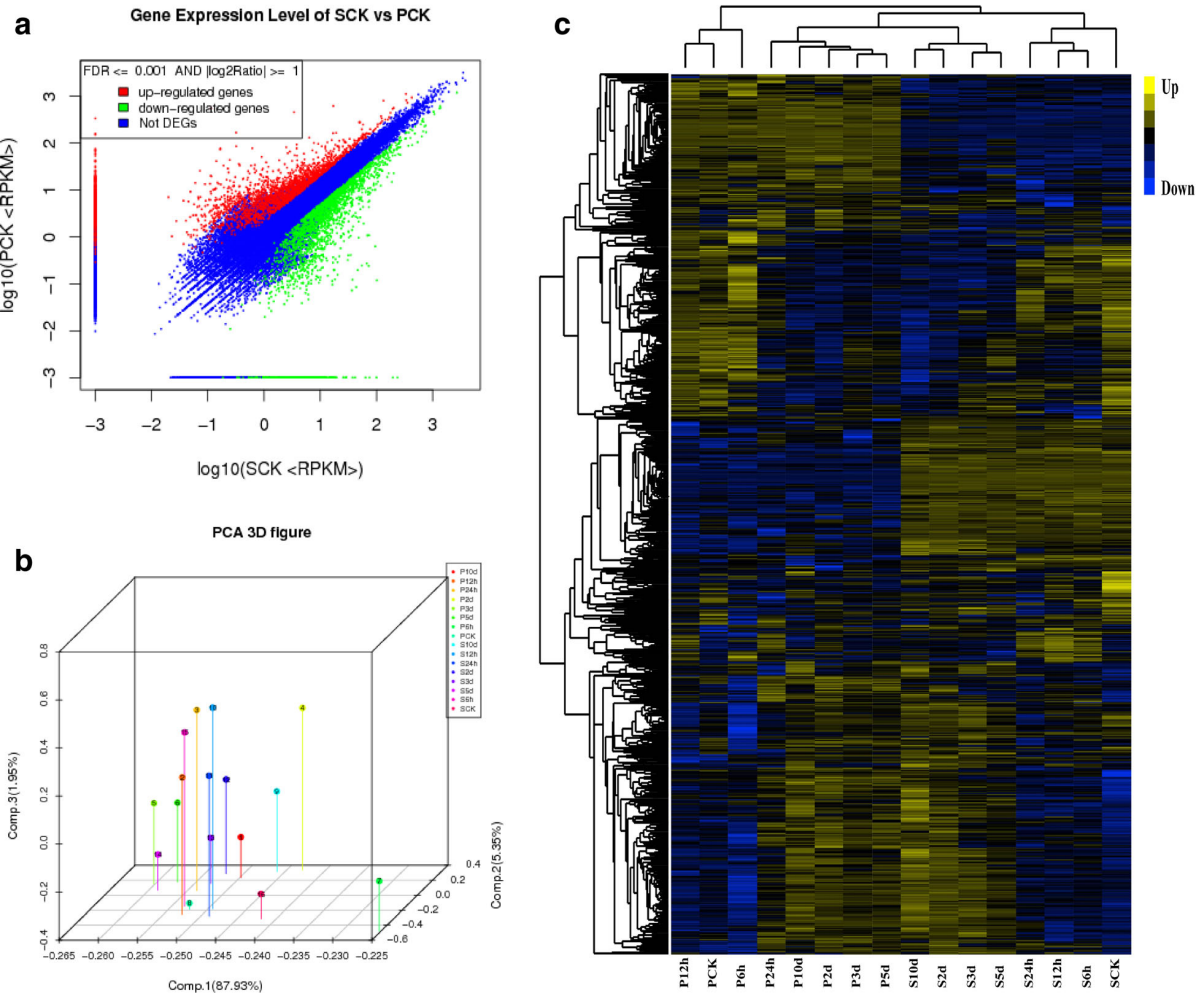
Comparison of resistant genotype (S18) and susceptible genotype (P77) in response to *V. dahliae* inoculation. **a** Scatter plot of differentially expressed genes (DEGs) in the uninoculated resistant genotype (SCK) and susceptible genotype (PCK). **b** PCA3D figure, each dot represents a sample on the principal component value. **c** Hierarchical clustering of 2107 DEGs; the signal ratios are shown in a *yellow-blue* color, where *yellow* represents up-regulation and *blue* represents down-regulation. Each column (PCK, P6h, P12h, P24h, P2d, P3d, P5d, P10d and SCK, S6h, S12h, S24h, S2d, S3d, S5d, S10d) represents the RPKM value in each sample and each row represents DEGs. S and P: S18 and P77; CK: the genotype exempt from pathogen stress as control; 6 h, 12 h, 24 h, 2d, 3d, 5d, and 10d: resistant genotype (S18) and susceptible genotype (P77) infected with *V. dahliae* after 6, 12, 24 h and 3, 5, 10 days respectively

In the present study, we performed transcriptome analysis of 16 samples (Fig. 2b and c) to describe the sunflower root response to SVW, and 509,533,702 reads were sequenced for library establishment. The length of most of the reads were distributed between 125 and 175 bp. Thus, we used the short reads comparison software TMAP [20] to compare reads with the reference gene sequences (allowing two-base mismatches), obtaining a total of 492,889,748 mapped reads (96.73% of the total reads) (Additional file 2: Table S1). Moreover, we obtained a total of 76,011 unigenes, with a mean length of 890 bp. The size distribution showed that 33,287 (43.8%) unigenes ranged in size from 200 to 500 bp, 19,344 (25.4%) unigenes ranged in size from 500 to 1000 bp, and 30.8% unigenes showed sizes greater than 1000 bp.

The expression level for each gene was calculated using the RPKM [21] method (Reads Per kb per Million reads). The related information for each gene (coverage, symbol, description, etc.) was also provided. To gain insight into the functions of the genes with responses to SVW, all unigenes were annotated using WEGO [22] and Blast2GO [23] software and were classified into functional categories. KEGG Pathway significant enrichment analyses were also performed for all genes. Specific expression analysis [24] was also used in the present study (Additional file 1: Figure S2). Scatter plot, principal component and cluster analyses were also used to mine the deep relation of different samples (Fig. 2a, b and c). These results revealed a large difference between the resistant and susceptible genotypes.

### Inter-genotypes differences in basal gene expression

The uninoculated resistant (S18) and susceptible (P77) genotypes were further compared to analyze the basal gene expression pattern (Fig. 2a). Differential expression analysis revealed 4937 significantly DEGs based on the False Discovery Rate Value (FDR) < = 0.001 and the absolute value of fold-change (FC) > = 2 (Additional file 3: Table S2). Moreover, among all DEGs, 1057 genes had a much higher expression value for the resistant genotype, with RPKMs ranging from 15 to 2685, and a total of 962 genes had a much higher expression value for the susceptible genotype, with RPKMs ranging from 15 to 1194. In particular, 358 genes were expressed at low level in the susceptible genotype (FC <0.001), but they had a higher expression value in the resistant genotype (FC > = 10). As inter-genotype differences may reveal the mechanisms of disease resistance and susceptibility [25], we also classified these DEGs according to functional categories. Following the Nr annotations, the DEGs were mapped into three GO categories, including 58 sub-categories (Additional file 1: Figure S3). Moreover, KEGG analyses were performed to identify the basal level biological pathways in sunflower roots. All DEGs were enriched into 120 KEGG pathways. Moreover, 16 important pathways were obtained with *P* values < =0.01 (Additional file 4: Table S3, Additional file 1: Figure S4).

### Transcriptional changes in response to *V. dahliae* inoculation

After the multiple comparisons (Additional file 1: Figure S5), two criteria of DEGs were defined: |FC value| > = 2 with *P*-value < = 0.01, and FDR < = 0.001 [26]. The results of the differential comparison between control and inoculated samples are shown in the RD and SD datasets (Additional file 5: Table S4-6). The 14 datasets represented the DEGs in response to SVW for each genotype. The comparison results between two inoculated samples for resistant and susceptible genotypes at same time point is shown in the D datasets, representing the DEGs between resistant and susceptible genotypes in response to SVW infection (Additional file 5: Table S4–6).

The dynamic changes with time of the DEGs in RD, SD and D datasets were also investigated (Additional file 1: Figure S6). The results showed that the remarkable changes in the transcriptome profile occurred on day 2. The number of DEGs in the D datasets, including both total and up-regulated DEGs, was markedly more than those in other two datasets at the first three time points. For the RD datasets (total, up or down-regulated DEGs), the numbers of DEGs was much more than that in the SD datasets at the last three time points (3, 5, and 10 d). In contrast, the numbers of DEGs in the SD datasets have relatively fewer changes than the other two datasets at the last three time points (3, 5, and 10 d).

**Fig. 3:**
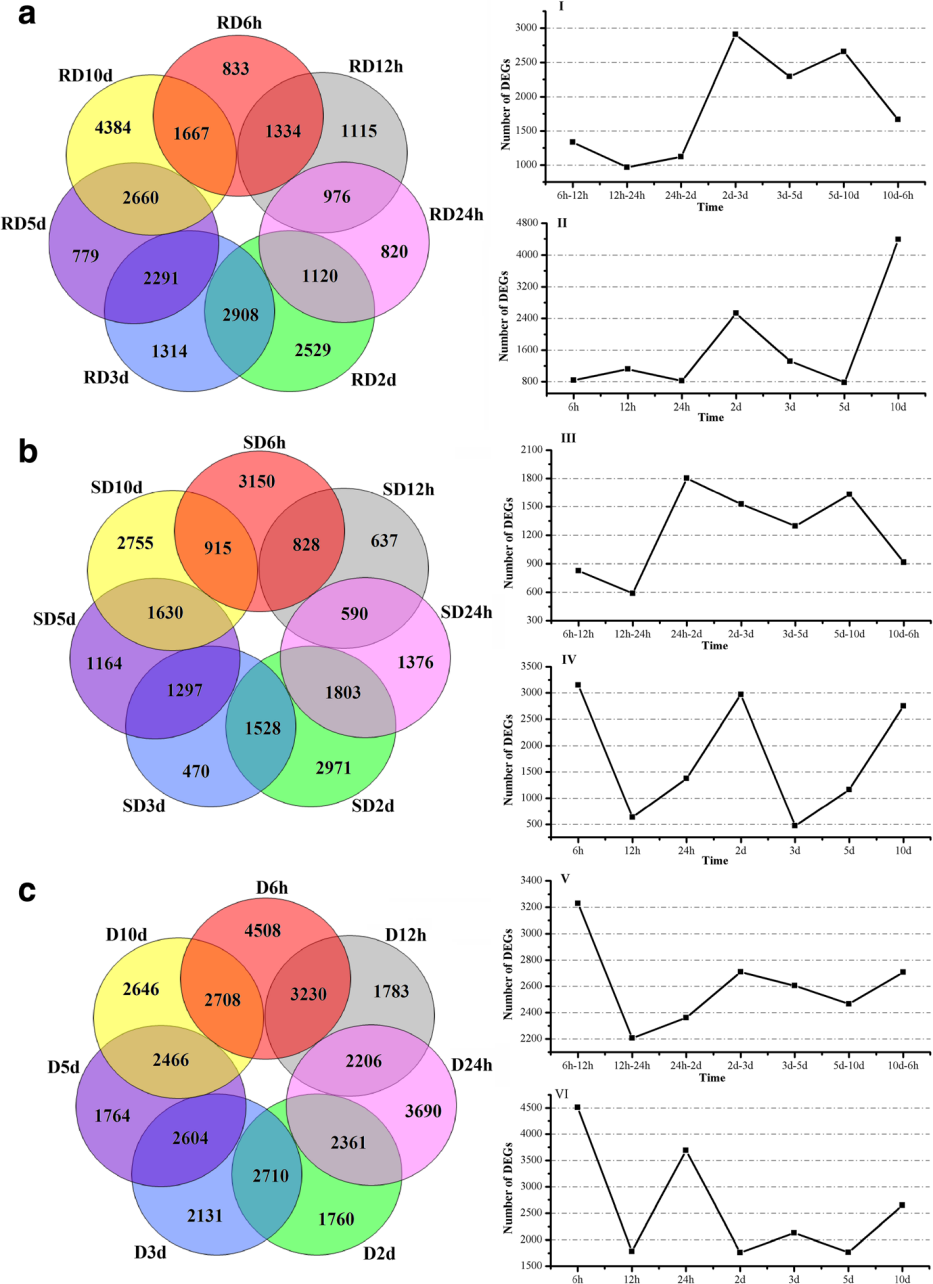
The distribution of DEGs between neighboring datasets. The numbers of DEGs exclusively regulated in each dataset are shown in the circle. The numbers of the time-common DEGs are shown in the overlapping regions. The time-specific DEGs are shown in the remaining regions. **a** and **b** represented DEGs of the RD and SD datasets; **c** represented DEGs of D datasets. I, III, and V represented change of the time-common DEGs; II, IV and VI represented the change of the time-specific transcription

Further, time-common DEGs and time-specific DEGs were examined (Fig. 3a, b, c). The common DEGs between neighboring time points were defined as time-common DEGs. The specific DEGs for their own datasets were defined as time-specific DEGs. The time-common DEGs showed more overlap in the datasets of 2–3 d in RD and 24 h-2 d in SD (Fig. 3I, III). For D datasets, the time-common DEGs were more common in neighboring data than in the RD and SD datasets (Fig. 3I, III, V). And, Fig. 3II, IV and VI show the trend of time-specific DEGs.

### Genotype-common transcriptional changes in response to *V. dahliae* inoculation

**Fig. 4:**
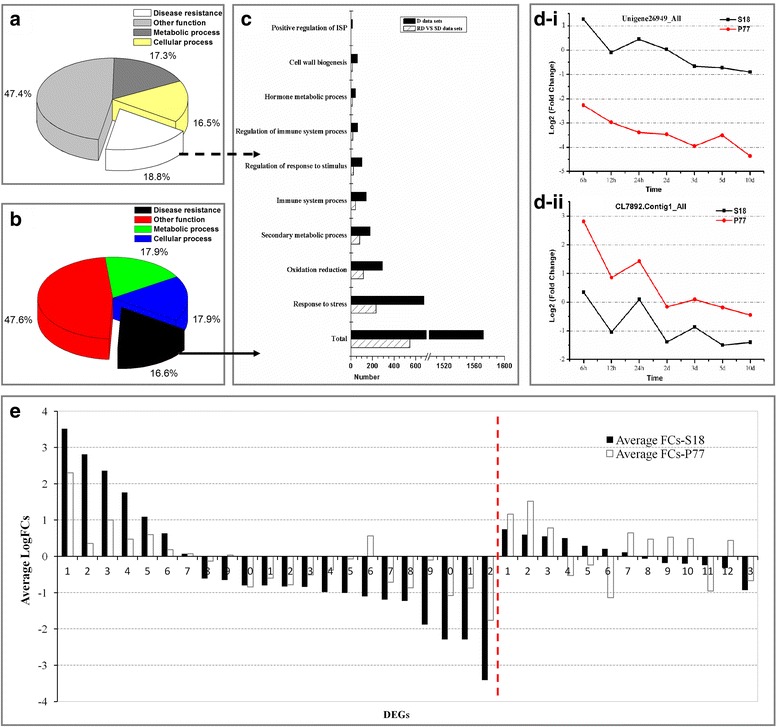
Genotype-common or genotype-specific transcriptional changes in DEGs in the resistant genotype (S18) and the susceptible genotype (P77). **a** Distribution of genotype-common transcriptional DEGs, modulated in RD and SD datasets into functional categories. **b** Distribution of genotype-common transcriptional DEGs, modulated in D datasets into functional categories. **c** Distribution of the genes involved in disease resistance, modulated in both genotypes into functional categories. **d** The dynamic trends of two DEGs involved in disease resistance. **e** 22 genotype-specific transcriptional DEGs encoded peroxidase (POD) in S18, 13 genotype-specific transcriptional DEGs encoded peroxidase (POD) in P77. The left side of the red line represents variations in the average fold-changes in genotype-specific transcriptional DEGs in S18, while the right side of the red line represents that in P77

For the time-common DEGs, the property of the genes between RD and SD datasets was also investigated. A total of 1319 co-expressed DEGs were defined as genotype-common transcriptional DEGs (Additional file 6: Table S7). The functional categories of the largest percentages of the common genes were related to ‘metabolic process’ (17.3%) and ‘cellular process’ (16.5%), followed by ‘disease resistance’ (18.8%) (Fig. 4a). Among these disease resistance categories, the cluster ‘response to stress’ (234, 8.1%) represented the largest group, followed by ‘oxidation reduction’ (115, 3.9%); ‘secondary metabolic process’ (82, 2.8%); and ‘immune response’ (42, 1.4%). Others disease resistance categories, such as ‘hormone metabolic process’ , ‘positive regulation of immune system process’ , ‘regulation of response to stimulus’ and ‘cell wall biogenesis’ , were also studied (Fig. 4c).

The 5956 time-common DEGs in the D datasets were also co-expressed in both genotypes and were also observed as DEGs with genotype-common transcriptional pattern (Additional file 6: Table S7). The functional categories of the largest percentages of the common genes were related to ‘metabolic process’ (17.9%) and ‘cellular process’ (17.9%), followed by ‘disease resistance’ (16.6%), which included the categories: ‘response to stress’ (677, 6.5%); ‘oxidation reduction’ (292, 2.8%); ‘secondary metabolic process’ (178, 1.7%); and ‘immune response’ (142, 1.4%). Other disease resistance categories were also observed following detailed analysis (Fig. 4b, c).

After analyzing the genotype-common transcriptional pattern, 1231 genes related to SVW resistance were observed in the RD-VS-SD and D datasets (Additional file 6: Table S7, Additional file 7: Table S8). The genes encoding to peroxidase (POD), glutathione peroxidase, aquaporin PIP, chitinase, L-ascorbate oxidase, and LRR receptors were identified using GO enrichment (Additional file 7: Table S8). For example, unigene_26949, which encoded peroxidase, showed higher FCs at different time points in the resistant genotype. The inverse result was observed for gene CL7892, which encoded L-ascorbate oxidase (Fig. 4d). When we focused on the average fold-change of each DEG for each functional category in genotype-common DEGs, most of the up-regulated DEGs in the resistant genotype showed a higher average FC than the susceptible genotype, and several down-regulated DEGs in the resistant genotype displayed lower average FCs than the susceptible genotype (Additional file 7: Table S8).

### Genotype-specific transcriptional changes in response to *V. dahliae* inoculation

Certain time-common DEGs were unique to the resistant (S18) or susceptible (P77) genotypes and were defined as genotype-specific transcriptional DEGs. A total of 4112 and 3007 DEGs were observed as genotype-specific transcriptional DEGs in resistant and susceptible genotypes, respectively (Additional file 8: Table S9). The functional enrichment of DEGs showed that the largest percentages of the genes were related to ‘metabolic process’ (17.4 and 17.8%, respectively) and ‘cellular process’ (17.6 and 16.7%, respectively), followed by genes involved in ‘disease resistance’ (17.7 and 18.2%, respectively) (Additional file 1: Figure S7A and B). Among these disease resistance categories in the resistant and susceptible genotypes, most of the DEGs were involved in the cluster ‘response to stress’ (621 and 415, respectively); followed by ‘oxidation reduction’ (274 and 231, respectively); secondary metabolic process’ (177 and 142, respectively); and ‘immune response’ (135 and 74, respectively, Additional file 1: Figure S7C). We concluded that the resistant genotype expressed more disease-resistance genes than the susceptible genotype (Additional file 1: Figure S7).

Analysis of the genotype-specific transcriptional pattern revealed 759 and 511 genes directly related to SVW resistance in the resistant and susceptible genotypes, respectively (Additional file 9: Table S10). Transcripts related to peroxidase (POD), L-ascorbate oxidase, aquaporin PIP, LRR receptor-like serine and the proto-oncogene protein myb were identified using GO enrichment. A total of 22 genotype-specific transcriptional DEGs encoding peroxidase (POD) were identified in the resistant genotype, while only 13 DEGs were identified in the susceptible genotype. Remarkably, for up-regulated genotype-specific DEGs in the resistant genotype, a higher average FC was observed in the resistant genotype than in the susceptible genotype (Fig. 4e, left part of red line). The down-regulated genotype-specific DEGs in the resistant genotype showed a lower average FC (Fig. 4e, left part of red line). Similar results were observed in genotype-specific transcriptional DEGs encoding L-ascorbate oxidase, aquaporin PIP, LRR receptor-like serine kinase and the myb proto-oncogene protein (Additional file 10: Table S11). However, no similar pattern was observed in the susceptible genotype (Fig. 4e, right part of red line). This conclusion was consistent with the results of the analysis of POD physiological indexes in sunflower roots post inoculation (Fig. 1).

### Validation of RNA-Seq data by quantitative real-time PCR

**Fig. 5:**
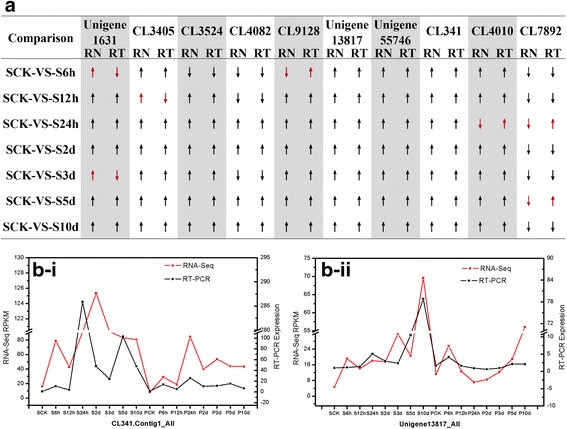
Comparison of RNA-Seq and the quantitative real-time PCR analyses for gene expression validation. The up arrow indicates the up-regulation of DEGs, the down arrow indicates the down-regulation of DEGs. RN indicates RNA-Seq, RT indicates qPCR. **a** Comparison of RNA-Seq and qPCR analyses for the genes expression validation in RD datasets. **b** The expression validation of DEGs (CL341.Contig1_All and Unigene 13817_ALL) using qPCR

To validate the RNA-Seq expression profiles of DEGs, the quantitative real-time PCR (qPCR) of three independent replicatates was performed. A total of 18 DEGs were randomly selected from the comparison between the uninoculated resistant and susceptible genotypes to validate the RNA-Seq expression profiles, and 14 DEGs detected using qPCR were consistent with the RNA-Seq data (Additional file 1: Figure S8). Further, we randomly selected 10 DEGs from RD and SD datasets (Additional file 11: Table S12). For RD datasets, 90.0% (63/70) of the qPCR results were consistent with the RNA-Seq data (Fig. 5a). For SD datasets, 84.3% (59/70) of the qPCR results were consistent with the RNA-Seq data (Additional file 1: Figure S9).

Generally, most of the genes assayed in the inoculated resistant genotype showed higher expression levels, and they were further analyzed by RNA-Seq and confirmed using qPCR, consistent with results of studies of the major physiological indexes in sunflower roots post inoculation (Fig. 5b-i and ii).

### Identification of novel *V. dahliae* inoculation-responsive genes

By analyzing the inter-genotype differences in basal gene expression, genotype-common transcriptional changes and genotype-specific transcriptional changes in response to *V. dahlia* inoculation, showed 2107 DEGs (Additional file 12: Table S13), which were related to SVW resistance. The results of cluster analysis for the DEGs showed that the expression patterns of the DEGs significantly varied in response to *V. dahliae* in both genotypes at different time points. Generally, similar expression patterns were observed among adjacent time point samples in the same genotype. In both resistant and susceptible genotypes, the samples possessed more up-regulated genes 2 days after inoculation. Interestingly, Some DEGs were up-regulated in the resistant genotype and down-regulated in susceptible genotype, while other DEGs showed the opposite result (Fig. 2c).

Interestingly, in the interaction mechanism of Verticillium wilt and sunflower, for 2107 DEGs, KEGG enrichment analyses were performed to identify the biological pathways in sunflower root. The results showed that 112 unigenes involved in KEGG pathway related to plant-pathogen interaction, which regulate 35 crucial points (Additional file 1: Figure S10); 97 DEGs were involved in KEGG pathway related to plant hormone signal transduction, which regulate 20 crucial proteins (Additional file 1: Figure S11); 53 DEGs were associated with KEGG pathway related to flavonoid biosynthesis, which regulate 7 critical points (Additional file 1: Figure S12); and 7 DEGs were involved in the KEGG pathway related to benzoxazinoid biosynthesis (Additional file 13: Table S14, Additional file 1: Figure S13).

Based on plant-pathogen interaction pathway, we observed that the hyper-sensitive response (HR) was regulated through the reactive oxygen species (ROS) and oxide (NO) signaling pathways. Respiratory burst oxidase homolog (RBOH) and flagellin-sensitive 2 (FLS2) could activated ROS. Cyclic nucleotide-gated ion channel (CNGC) could mediate the NO signaling pathway (Additional file 1: Figure S14A). In the present study, three genes (Unigene21981_All, Unigene20875_All and Unigene11922_All) encoding RBOH were up-regulated (average FC > 0) in both genotypes, based on the comparison between treated and untreated sunflower genotypes. Notably, the expression levels of the genes in the resistant genotype were highly increased by comparing with those in susceptible genotype after pathogen challenge. However, most of the genes encoding FLS2 were down-regulated in both the resistant or susceptible genotypes after inoculation, and the expression levels of the genes in the resistant genotype were highly decreased by comparing with those in the susceptible genotype. Perhaps to compensate for the loss, the expression levels of three genes in the resistance genotype were more increased. Furthermore, the results showed that the genes encoding CNGC were down-regulated or up-regulated in both genotypes after the infection. However, expression levels of some of genes were more enhanced in the resistant genotype. Remarkably, four genes encoding CNGC were up-regulated in the resistant genotype, but were down-regulated in the susceptible genotype (Additional file 13: Table S14). In plant hormone signal transduction pathway, the dissociation between JAZ (jasmonate ZIM domain-containing protein) and the transcription factor MYC2 could induce defense-related genes, and was initiated by jasmonic acid (JA). This dissociation could be activated by COI1 (Additional file 1: Figure S14B). In this study, the genes (e.g., CL200.Contig5_All) related to COI1 were promoted in the resistant genotype, but were enhanced in the susceptible sunflower genotype when compared treated with untreated sunflowers. Four genes encoding JAZ were up-regulated in the susceptible sunflower genotype, but two genes (Unigene15789_All, CL9889.Contig2_All) were down-regulated in the resistant sunflower genotype. For the salicylic acid (SA)-mediated signal transduction pathway, to induce the expression of defense genes, such as the pathogenesis-related gene PR-1 (pathogenesis-related protein 1), NPR1 must activate the activity of transcription factor TGA. In the present study, Unigene28388_All, which encoded NPR1, was up-regulated in the resistant sunflower genotype, but were down-regulated in the susceptible sunflower genotype. Four genes encoding TGA were up-regulated in the resistance sunflower genotype. Notably, the expression levels of the genes in the resistant genotype were remarkably increased when comparing them with those in the susceptible genotype (Additional file 13: Table S14).

**Fig. 6:**
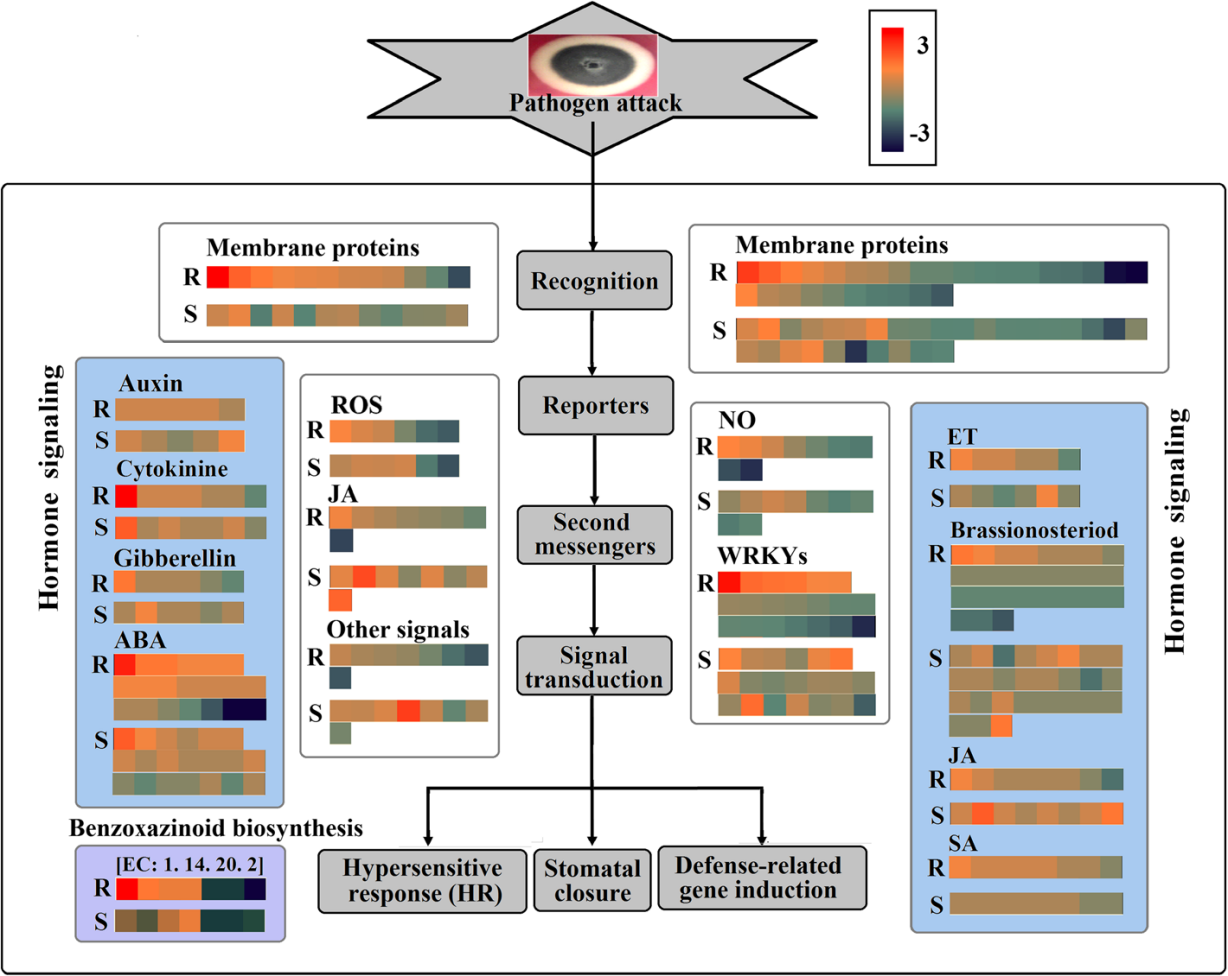
Distribution of differentially expressed genes specific to the resistant genotype and the susceptible genotype in response to *V. dahlia* infection. Each square represents the fold-change of one gene in the resistant (S18) or susceptible (P77) genotypes, where *red* represents up-regulation and deep *blue* represents down-regulation

Furthermore, we identified 20 ABA (abscisic acid)-related genes, among which 14 genes were up-regulated in the resistant genotype compared with the susceptible genotype, and 3 DEGs showed distinct down-regulation in the resistant genotype compared with the susceptible genotype. In addition, we identified 8 JA-related genes and 6 ET-related genes (Fig. 6). All these genes were well represented in both genotypes and a higher average FC in the resistant genotype than in the susceptible genotype was observed for many of the up-regulated genes, while a lower average FC in the resistant genotype than in the susceptible genotype was observed for many of the down-regulated genes (e.g. the DEGs encoded membrane proteins, NO and WRKYs), confirming the results of genotype-specific transcriptional pattern analysis (Fig. 6, Additional file 12: Table S13). In particular, these down-regulated genes may regulate important signaling pathway. This conclusion was consistent with the research results of Sun et al., revealing that the down-regulation of GhCYP82D might regulate the JA signaling pathway [9].

## Discussion and conclusions

Plants adapt to complex environments by defending themselves against a wide range of pathogens that have different lifestyles [27]. To reveal how plants resist infections, RNA-Seq was used in the present study. A total of 76,011 unigenes were obtained. We focused on the genes associated with disease resistance. Previous studies have shown that peroxidases, aquaporins and chitin, which are widely distributed in the plant kingdom [28–31], are activated in defense responses against pathogens [32–36]. In addition, ascorbate oxidase (AO) is a potential conductor of a symphony of signaling pathways [37], AO over expression or suppression was apparently influenced under unfavorable environmental conditions [38–41]. Several studies on LRR disease resistance proteins confirmed an association with plant resistance to fungal diseases [32, 42]. In present study, we analyzed the genotype-common transcriptional pattern, identifying 1231 genes related to SVW resistance in the RD-VS-SD and D datasets; analysis of the genotype-specific transcriptional pattern, showed 759 and 511 genes directly related to SVW resistance in the resistant and susceptible genotypes, respectively. Furthermore, the transcripts were analyzed using GO (Gene Ontology) classification analysis. Most of the genes were related to peroxidase (POD), glutathione peroxidase, aquaporin PIP, chitinase, L-ascorbate oxidase, and LRR receptors. Remarkably, for up-regulated genotype-specific DEGs in the resistant genotype, a higher average FC in the resistant genotype was observed compared with the susceptible genotype and the inverse was observed in down-regulated genotype-specific DEGs in the resistant genotype. This conclusion indirectly confirmed the results of the study on the main physiological indexes in sunflower roots.

For resistance to *V. dahlia* infection, sunflowers induce the HR and cell wall reinforcement. The HR is activated through ROS, JA, WRKYs and the NO signaling pathway [12, 43] and is mediated by CNGC, RBOH, CaM/CML and FLS2 [44–46]. In the present study, the comparison between treated and untreated sunflower genotypes showed that most of the genes encoding RBOH and CNGC were up-regulated in the both genotypes, and most of the genes encoding FLS2 were down-regulated in both genotypes. Furthermore, the expression levels of up-regulated genes were more highly increased and expression levels of the down-regulated genes were more highly decreased in the resistance genotype.

In plants facing pathogen infection, a variety of hormonal signaling pathways involved in defense against all types of challenges were induced [47–49]. Jasmonates and SA are essential phytohormones for plant development and survival, and play an important role in plant resistance to fungal diseases [50, 51]. COI1 is a key component of the JA-mediated signal transduction pathway, leading to the dissociation between JAZ and the transcription factor MYC2, which could induce defense-related genes. For the SA-mediated signal transduction pathway, NPR1 plays a critical role in the induction of defense genes by activating the transcription factor TGA [52, 53]. In the present study, the genes encoding JAZ were up-regulated in the susceptible sunflower genotype, but two of these genes were down-regulated in the resistant sunflower genotype. The COI1 and NPR1 and TGA genes were promoted in the resistant genotype but repressed in the susceptible sunflower genotype when comparing treated and untreated sunflower. Furthermore, the expression levels of the genes in the resistant genotype were remarkably increased compared with those in the susceptible genotype.

Furthermore, because sunflower has a large genome, studies have not previously elucidated any comprehensive sequence information to describe the transcriptome associated with defense responses against *V. dahliae*. Here, an RNA-Seq approach was employed to investigate the molecular interaction between sunflower and *V. dahliae.* The results showed that some of the genes related to SVW resistance involved in the plant-pathogen interactions and plant hormone signal transduction, flavonoid biosynthesis and benzoxazinoid biosynthesis pathways, which will strongly contribute to a better understanding of the molecular interactions between sunflower and *V. dahliae* and provide insights into the interaction between plants and pathogens. Accordingly, the findings of the present study will accelerate research on the resistance of sunflower to *V. dahliae* and contribute to a better understanding of the sunflower defense response to plant pathogens.

## Methods

### Plant materials and *V. dahliae* inoculation procedures

One highly aggressive strain of the defoliating fungus *V. dahliae*, V21, from Inner Mongolia Agricultural University Agricultural College, was used for inoculation. Two sunflower genotypes, the resistant genotype (S18, from Need Association of the Inner Mongolia Autonomous region) and the susceptible genotype (P77, from the Institute of Crop Breeding and Cultivation of Inner Mongolia Academy of Agricultural & Animal Husbandry Sciences), were used for inoculation. The seeds were treated with 70% ethanol for 5 min, and subsequently immersed in HgCl_2_ for 8–10 min to sterilize the surface, followed by rinsing three times with sterile distilled water.

The seeds of the resistant genotype and the susceptible genotype were grown in sterilized soil (a mix of peat and sawdust) in sterile culture pots at 25 °C. Each seedling was inoculated with 50 mL of *V. dahliae* spore suspension with 2 × 10^6^ spores/ml at the two-true-leaf growth stage [54]. Control plants were not inoculated but were treated and sampled with distilled water in the same manner. The root tissues from inoculated and non-inoculated plants were harvested for each treatment at each sampling time point (including 6, 12 and 24 h and 2, 5 and 10 d after inoculation), followed by washing with 75% alcohol and sterile water and immediately storing in liquid nitrogen.

### RNA extraction and cDNA preparation for Illumina sequencing

For samples of two genotypes, the RNA was isolated from sunflower roots using Trizol reagent (Invitrogen, US) according to the manufacturer’s instructions. The RNA samples were numbered with the corresponding sampling time points, i.e., S6h, S12h, S24h, S2d, S3d, S5d, and S10d for the inoculated resistant genotype (S18) and P6h, P12h, P24h, P2 d, P3d, P5d, and P10d for the inoculated susceptible genotype (P77). For the control plants were denoted as SCK and PCK (Fig. 2b, c). The total RNA samples were first treated with DNase I to remove any potential DNA contamination. Subsequently, the products were purified using magnetic beads and the mRNAs were enriched using oligo (dT) magnetic beads. These beads were mixed with fragmentation buffer, and the mRNAs were fragmented into short fragments (approximately 200 bp). Subsequently, first-strand cDNA was synthesized using random hexamer-primed reverse transcription. Buffer, dNTPs, RNase H and DNA polymerase I were added to synthesize second-strand cDNA. The double-standed cDNA was purified using magnetic beads. End reparation was subsequently performed. After the previous step, adaptors were ligated to the ends of these fragments. Next, ligation products were selected according to size and purified on TAE-agarose gels. Finally, the fragments were enriched through PCR amplification, purified using magnetic beads and dissolved in the appropriate amount of Epstein-Barr solution. During the QC step, an Agilent 2100 Bioanalyzer was used to qualify and quantify of the sample library. The libraries were sequenced using an Ion Proton sequencer when necessary.

### Functional annotation of unigenes using bioinformatics methods

We performed GO functional annotation using NR annotation. The basic unit of GO is the GO-term. Every GO-term belongs to a type of ontology. With NR annotation, we used the Blast2GO program for the GO annotation of unigenes. After obtaining a GO annotation for every Unigene, we used WEGO software for the GO functional classification of all unigenes and to understand the distribution of gene functions of the species at the macro level. Using the KEGG database, we further studied the biological complex behaviors of genes. We also conducted pathway annotation for the unigenes, and subsequently the unigenes were aligned to protein databases in the priority order of NR, Swiss-Prot, KEGG and COG using BLASTx (*E* value < 0.00001). The all-unigenes were assigned GO annotations using Blast2GO (http://www.blast2go.com/). In addition, unigenes were aligned with the NCBI nucleotide (Nt) databases using BLASTn with an *E* value of 1.0e^−5^.

### Quantitative real-time PCR expression analysis

Total RNA was extracted from the control and samples of resistant and susceptible genotypes as previously described. A 1-μg sample of total RNA reversed transcribed using M-MLV (M170A, Promega) was used for cDNA synthesis in a 20-μL reaction. A PCR instrument (Applied Biosystems 2720, USA) was used. A 1-μl sample of the single-strand cDNA determined using fluorimetric assay (QPK-201, TOYOBO) was used for qPCR in a 20-μL reaction on a quantitative real-time PCR Instrumen (BIO-RAD CFX-96). The 18S rRNA gene of sunflower was used as the reference gene [55]. Relative quantitative analysis was performed under the following conditions: 95 °C for 30 min, followed by 40 cycles of 95 °C for 10 s, 60 °C for 10s and 72 °C for 10s in a volume of 20 μl. A melting curve analysis at 95 °C for 15 s and 60 °C for 5 s was used to identify different amplicons, including non-specific products [12]. All reactions for each gene were performed in triplicate. The relative expression level of each gene among the samples was calculated using the 2 (−DeltaDeltaC(T)) method with normalization to the internal reference acting gene [56].

## Additional files

Additional file 1: Figure S1-S14. All supplementary Figures. (DOCX 2305 kb)
Additional file 2: Table S1. Statistics of the reads in resistant genotype (S18) and susceptible genotype (P77). (DOCX 16 kb)
Additional file 3: Table S2. DEGs of SCK-VS-PCK. (XLSX 1084 kb)
Additional file 4: Table S3. KEGG pathways of SCK-VS-PCK. (DOCX 16 kb)
Additional file 5: Table S4-S6. The summary of pairwise comparisons. (DOCX 17 kb)
Additional file 6: Table S7. Genotype-common transcriptional DEGs in RD-VS-SD and D datasets. (XLSX 4694 kb)
Additional file 7: Table S8. DEGs involved in SVW resistance in genotype-common pattern. (XLSX 53 kb)
Additional file 8: Table S9. Genotype-specific transcriptional DEGs results in resistant genotype and susceptible genotype. (XLSX 4357 kb)
Additional file 9: Table S10. DEGs related to SVW resistance in genotype-specific pattern. (XLSX 505 kb)
Additional file 10: Table S11. DEGs directly involved in SVW resistance in genotype-specific pattern. (XLSX 59 kb)
Additional file 11: Table S12. The raw data of q-PCR. (XLSX 78 kb)
Additional file 12: Table S13. All DEGs related to SVW resistance. (XLSX 814 kb)
Additional file 13: Table S14. The DEGs were associated with KEGG pathways. (XLSX 93 kb)

## Abbreviations

ABA: Abscisic acid
AO: Ascorbate oxidase
CNGC: Cyclic nucleotide-gated ion channel
DEGs: Differentially expressed genes
ET: Ethylene
FC: Fold-change
FDR: False Discovery Rate Value
FLS2: Flagellin-sensitive 2
GO: Gene Ontology
HR: Hypersensitive response
JA: Jasmonic acid
JAZ: Jasmonate ZIM domain-containing protein
MDA: Malondialdehyde
POD: Peroxidase
PR-1: Pathogenesis-related protein 1
qPCR: Quantitative real-time PCR
RBOH: Respiratory burst oxidase homolog
ROS: Reactive oxygen species
SA: Salicylic acid
SRA: Sequence Read Archive
SVW: Sunflower Verticillium wilt.
