# Supplementary material for: Large-scale transcriptome comparison of sunflower genes responsive to Verticillium dahliae
Source: BMC Genomics. 2017 Jan 6;18:42. doi: 10.1186/s12864-016-3386-7 (PMC5219742; doi:10.1186/s12864-016-3386-7)
Supplement: Additional file 1: Figure S1-S14. — All supplementary Figures. (DOCX 2305 kb) [file 12864_2016_3386_MOESM1_ESM.docx]

**Additional figure S1.** Dynamic of MDA in inoculated resistant genotype (S18) and susceptible genotype (P77) at different time points.


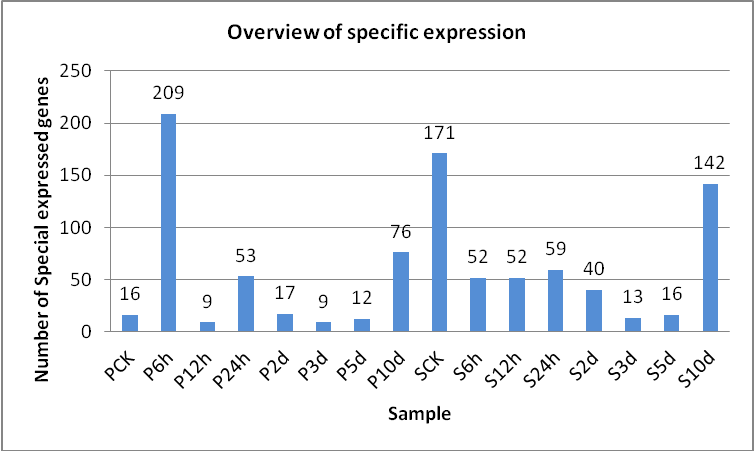


**Additional figure S2.** Overview of specific expression in resistant genotype (S18) and susceptible genotype (P77) at the different time points.


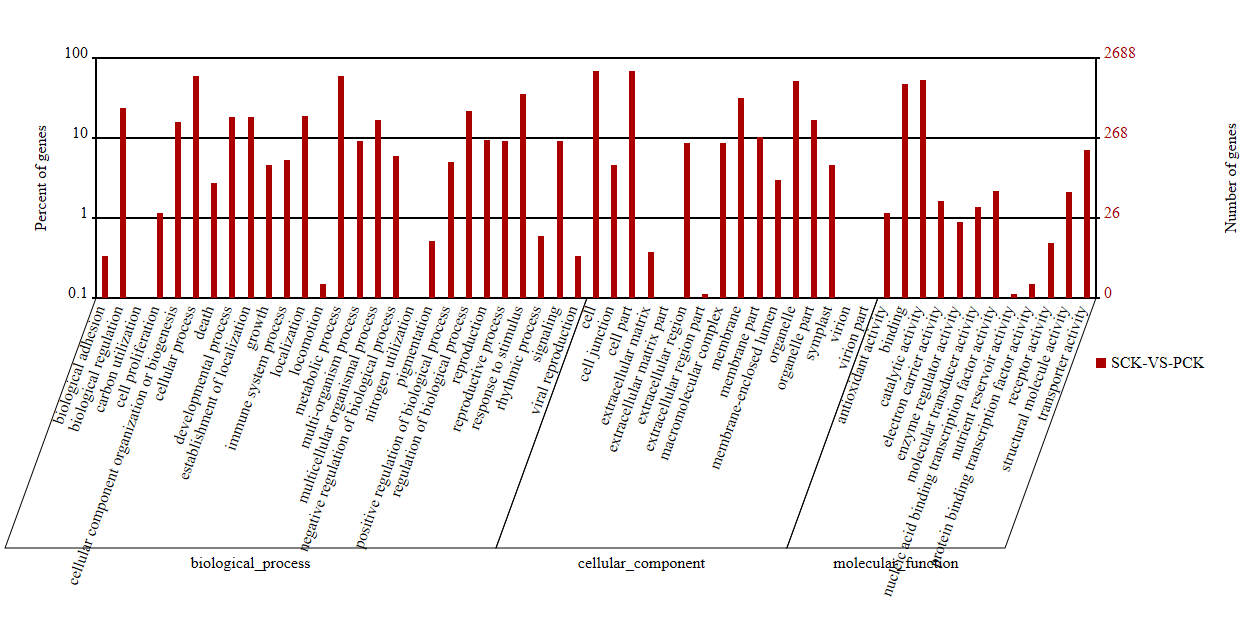


**Additional figure S3.** Gene Ontology functional classification of SCK-VS-PCK.


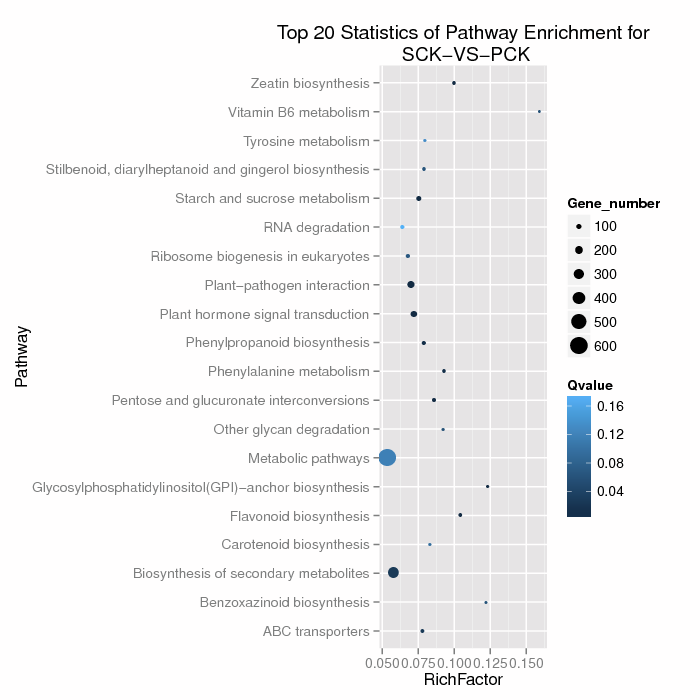


**Additional figure S4.** Scatter plot of KEGG pathway enriment statistics for SCK-VS-PCK.


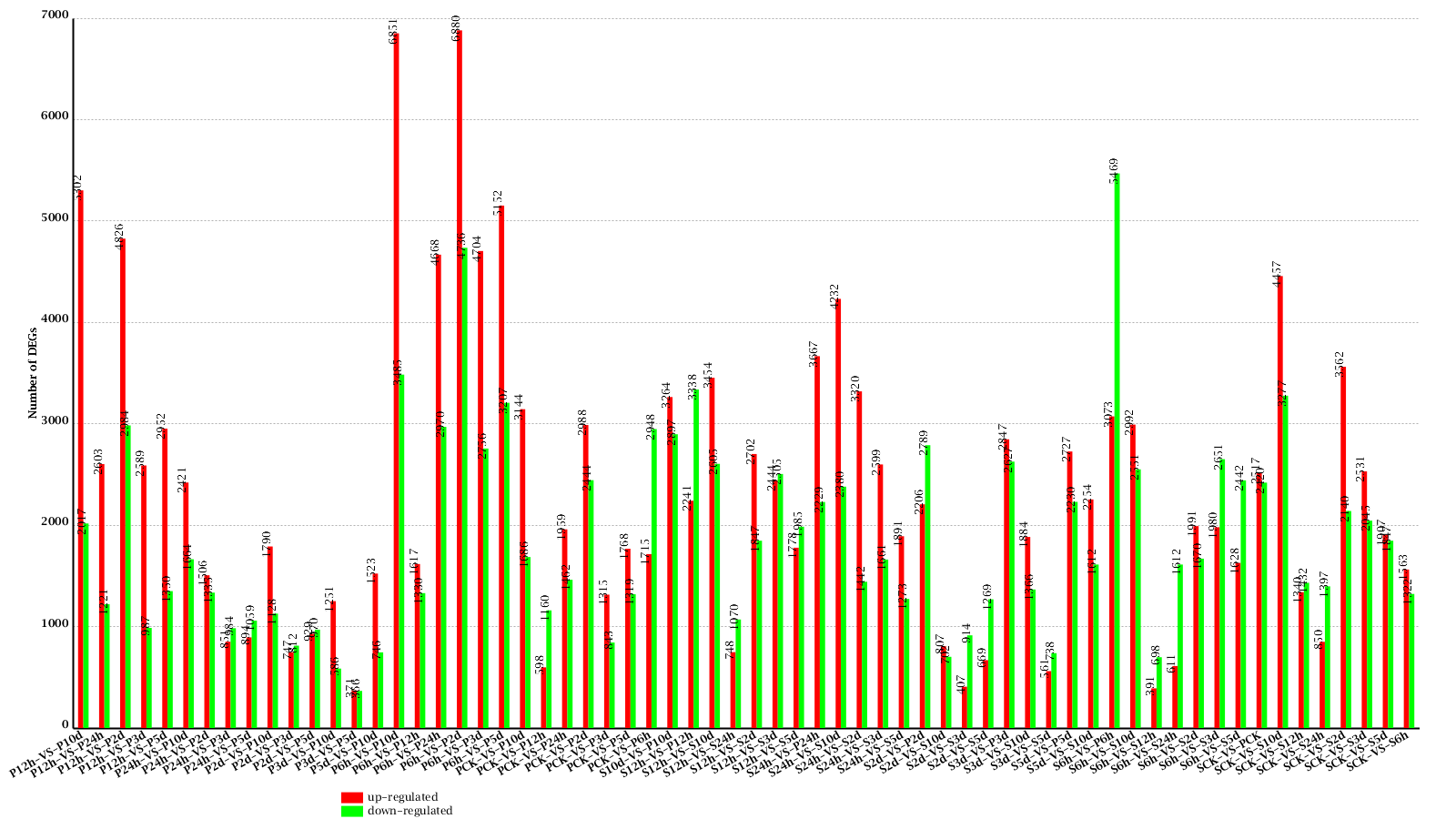


**Additional figure S5:** S**creening of differentially expressed unigenes (DEGs).** In a pairwise comparison (denote as A-VS-B for example), the former one (A) is considered as the control, and the latter one (B) is considered as the treatment.









**Additional figure S6 The dynamic changes of DEGs number at series time points.** The dynamic trends of DEGs number for total, up-regulated and down-regulated DEGs were showed in Figure A, B and C, respectively. The blue lings represent the RD data sets, and the red lines represent the SD data sets, the green lines represent the D data sets.


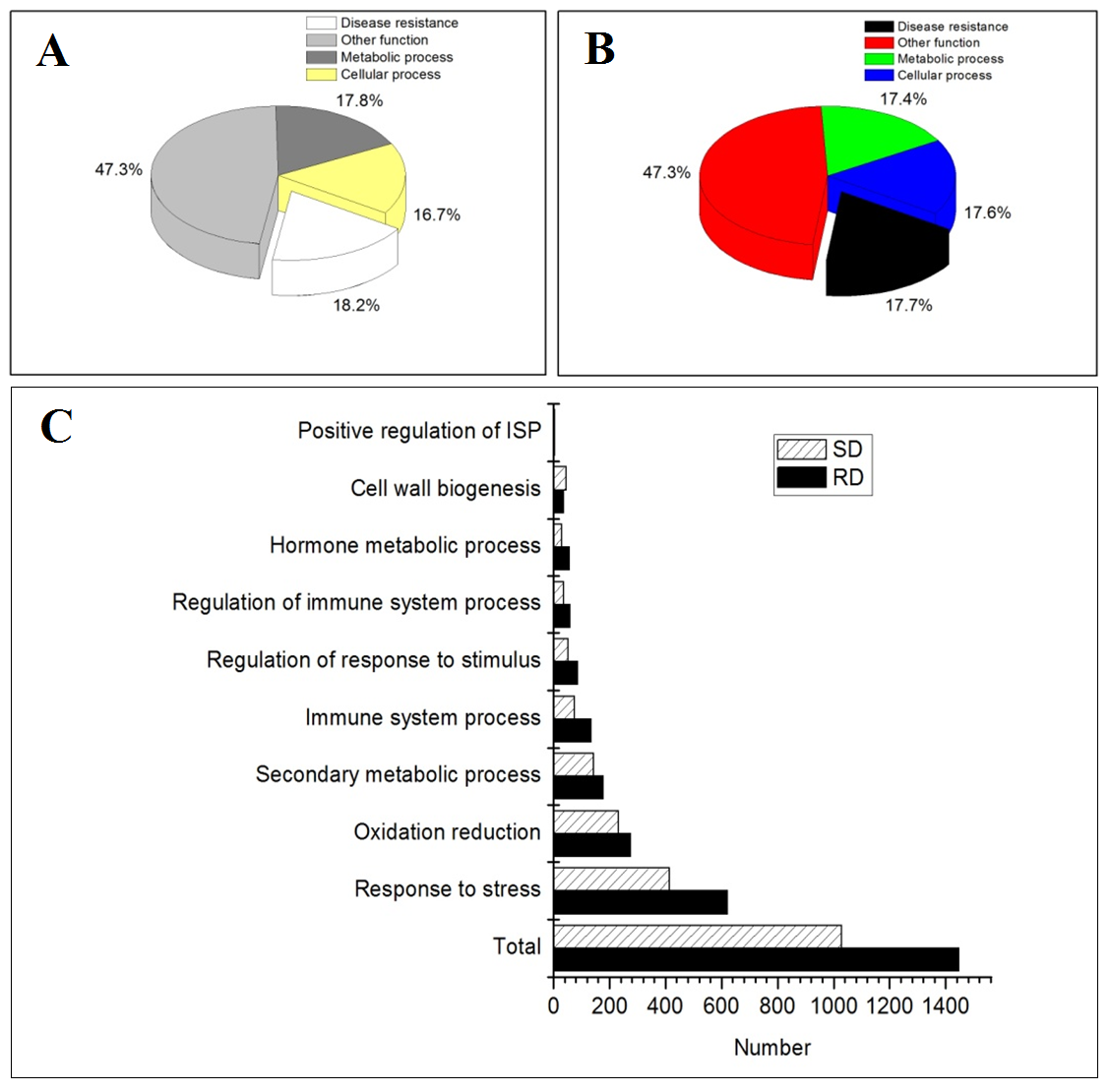


**Additional figure S7. Genotype-specific transcriptional changes in S18 and P77.** (A) Distribution of genes involved in SD data sets, modulated in both genotypes into functional categories. (B) Distribution of genes involved in RD data sets, modulated in both genotypes into functional categories. (C) Distribution of genes involved in disease resistance, modulated in both genotypes into functional categories.

**
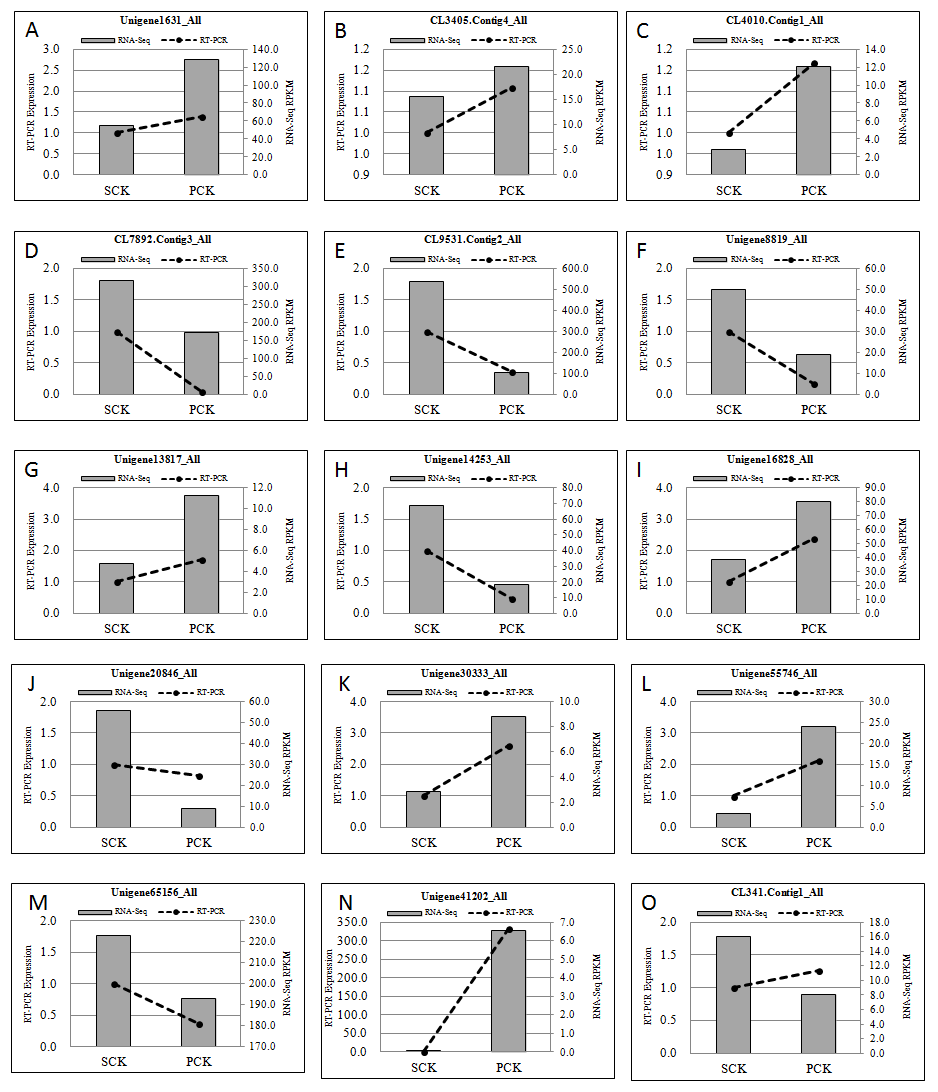
**

**Additional file Figure S8. Comparison of RNA-Seq and qRT-PCR analyses for basal gene expression validation.** Expression profiles of (A) heat shock protein 1 (GRMZM2G437100), (B) phospholipase D (Unigene1631_All), (C) tyrosine 3-monooxygenase(CL4010.Contig1_All), (D) L-ascorbate oxidase (CL7892.Contig3_All), (E) histone H1/5 (CL9531.Contig2_All), (F) calcium-binding protein CML (Unigene8819_All), (G) phospholipase (Unigene13817_All), (H) CCR4-NOT transcription complex subunit (Unigene14253_All), (I) aquaporin NIP (Unigene16828_All) (J) ath:AT5G22850 (Unigene20846_All l), (K) CREB-binding protein (Unigene30333_All), (L) catalase (Unigene55746_All) (M) flavonoid 3'-monooxygenase (Unigene65156_All), (N) translation initiation factor 3 subunit I (Unigene41202_All), (O) mandelonitrile lyase (CL341.Contig1_All ). RPKM values are represented by Histograms as assessed by RNA-Seq analysis, 2 (-DeltaDeltaC(T)) values are represented by dotted lines as assessed by RT-PCR analysis in SCK-VS-PCK.

| **Comparison** | **Unigene**  **1631** | | **CL3405** | | **CL3524** | | **CL4082** | | **CL9128** | | **Unigene**  **13817** | | **Unigene**  **55746** | | **CL341** | | **Unigene**  **14253** | | **Unigene**  **41202** | |
| --- | --- | --- | --- | --- | --- | --- | --- | --- | --- | --- | --- | --- | --- | --- | --- | --- | --- | --- | --- | --- |
|  | **RN** | **RT** | **RN** | **RT** | **RN** | **RT** | **RN** | **RT** | **RN** | **RT** | **RN** | **RT** | **RN** | **RT** | **RN** | **RT** | **RN** | **RT** | **RN** | **RT** |
| **PCK-VS-P6h** | **↑** | **↑** | **↑** | **↑** | **↓** | **↑** | **↑** | **↑** | **↓** | **↑** | **↑** | **↑** | **↓** | **↑** | **↑** | **↑** | **↓** | **↑** | **↑** | **↑** |
| **PCK-VS-P12h** | **↑** | **↑** | **↑** | **↑** | **↓** | **↑** | **↑** | **↑** | **↑** | **↑** | **↓** | **↓** | **↓** | **↑** | **↑** | **↑** | **↑** | **↑** | **↑** | **↑** |
| **PCK-VS-P24h** | **↑** | **↑** | **↑** | **↑** | **↑** | **↑** | **↑** | **↑** | **↑** | **↑** | **↓** | **↓** | **↑** | **↑** | **↑** | **↑** | **↑** | **↑** | **↑** | **↑** |
| **PCK-VS-P2d** | **↑** | **↑** | **↑** | **↑** | **↑** | **↑** | **↑** | **↑** | **↓** | **↑** | **↓** | **↓** | **↑** | **↑** | **↑** | **↑** | **↑** | **↑** | **↑** | **↑** |
| **PCK-VS-P3d** | **↑** | **↓** | **↑** | **↑** | **↑** | **↑** | **↓** | **↑** | **↑** | **↑** | **↑** | **↓** | **↓** | **↓** | **↑** | **↑** | **↓** | **↓** | **↑** | **↑** |
| **PCK-VS-P5d** | **↑** | **↑** | **↑** | **↑** | **↑** | **↑** | **↑** | **↑** | **↑** | **↑** | **↑** | **↑** | **↑** | **↑** | **↑** | **↑** | **↑** | **↑** | **↑** | **↑** |
| **PCK-VS-P10d** | **↑** | **↑** | **↑** | **↑** | **↑** | **↑** | **↑** | **↑** | **↑** | **↑** | **↓** | **↓** | **↑** | **↑** | **↑** | **↑** | **↑** | **↑** | **↓** | **↑** |

**Additional Figure S9.** Comparison of RNA-Seq and qRT-PCR analyses for the genes expression validation in SD data sets.


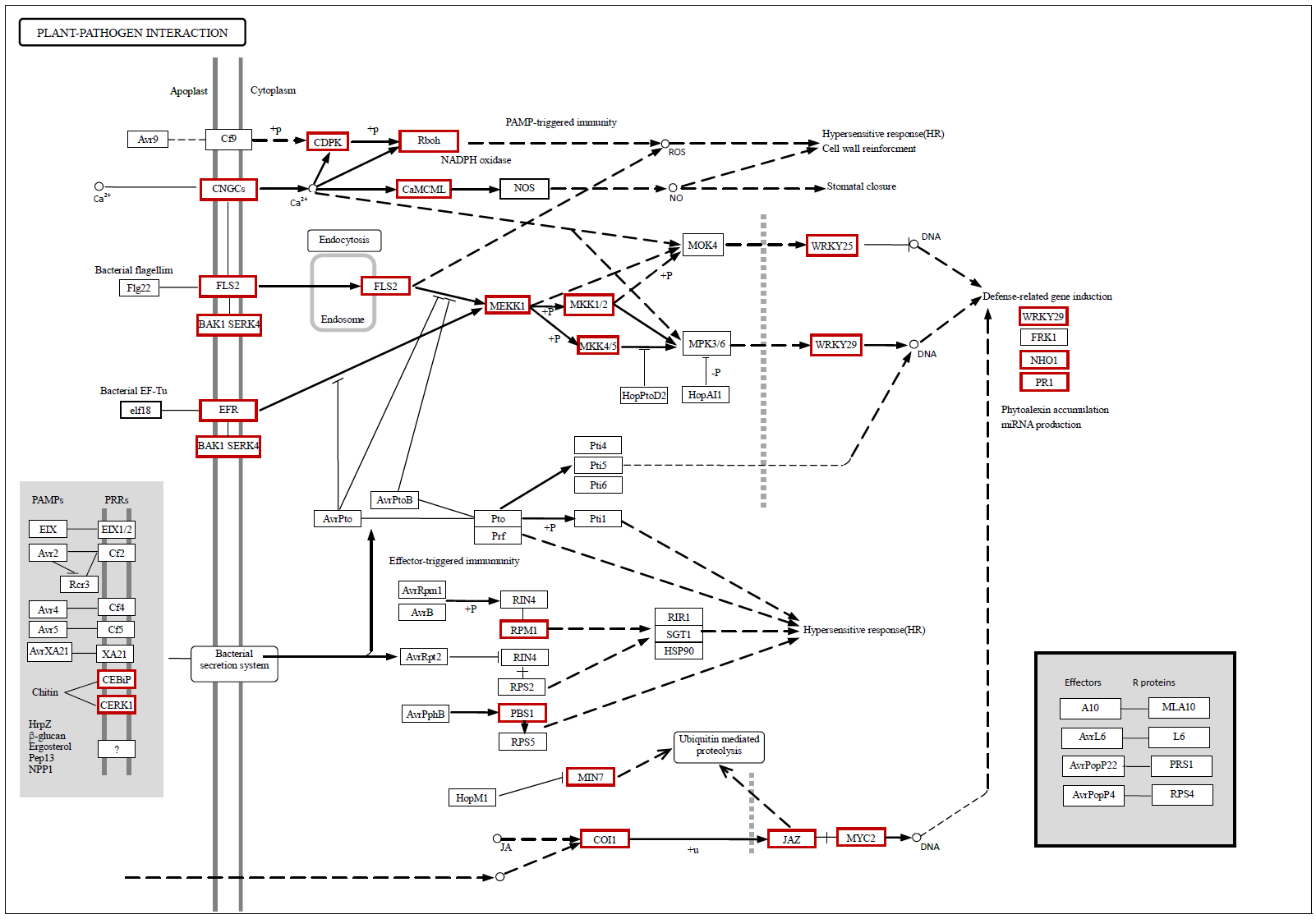


**Additional file Figure S10. Plant–pathogen interaction KEGG pathways.** Each box shows enzymes involved in the pathway. Genes highlighted in red were detected from the 2,107 DEGs.


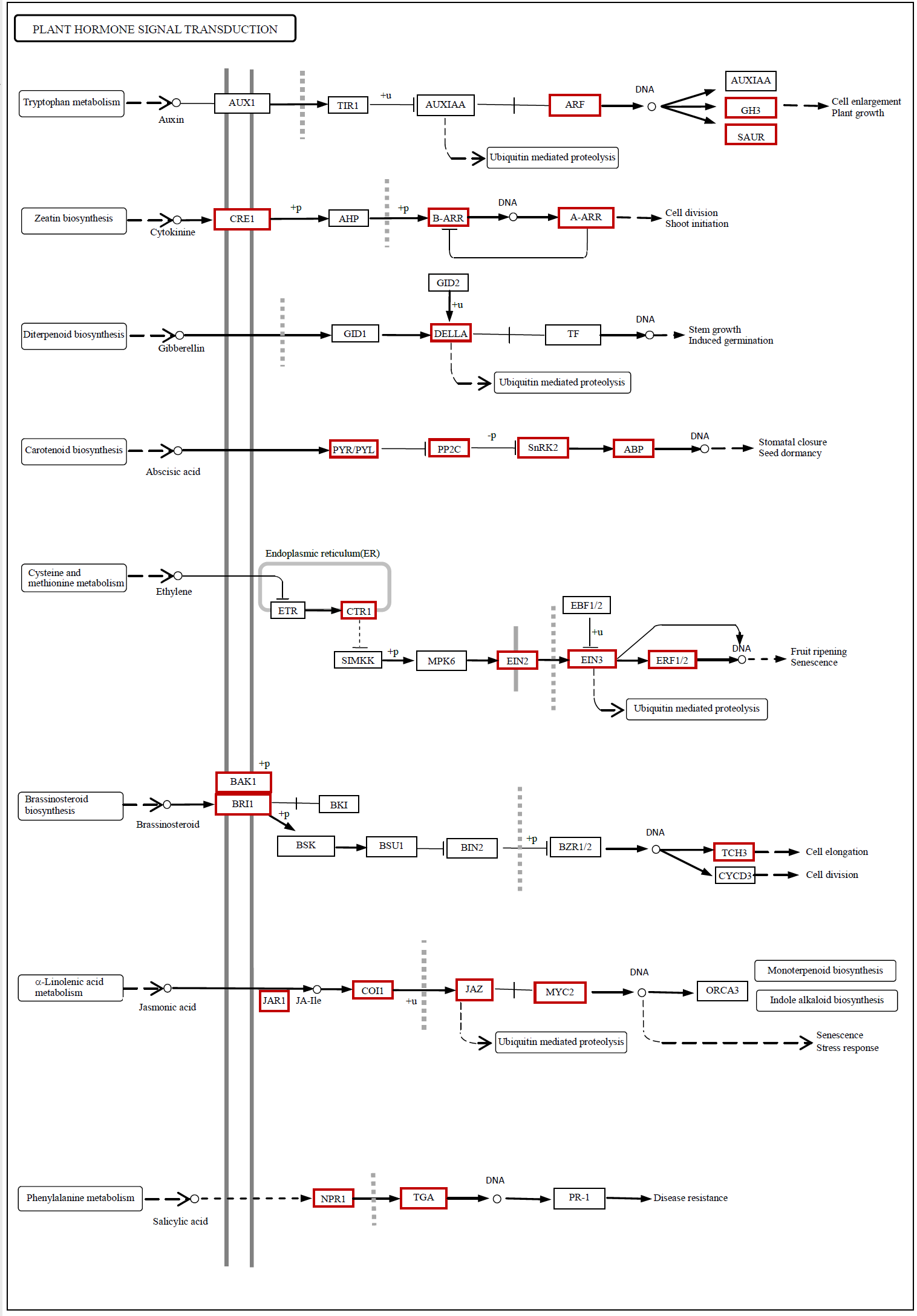


**Additional file Figure S11. Plant hormone signal transduction KEGG pathways.** The genes highlighted in red box shows enzymes involved in the pathway, which were detected from the 2,107 DEGs.


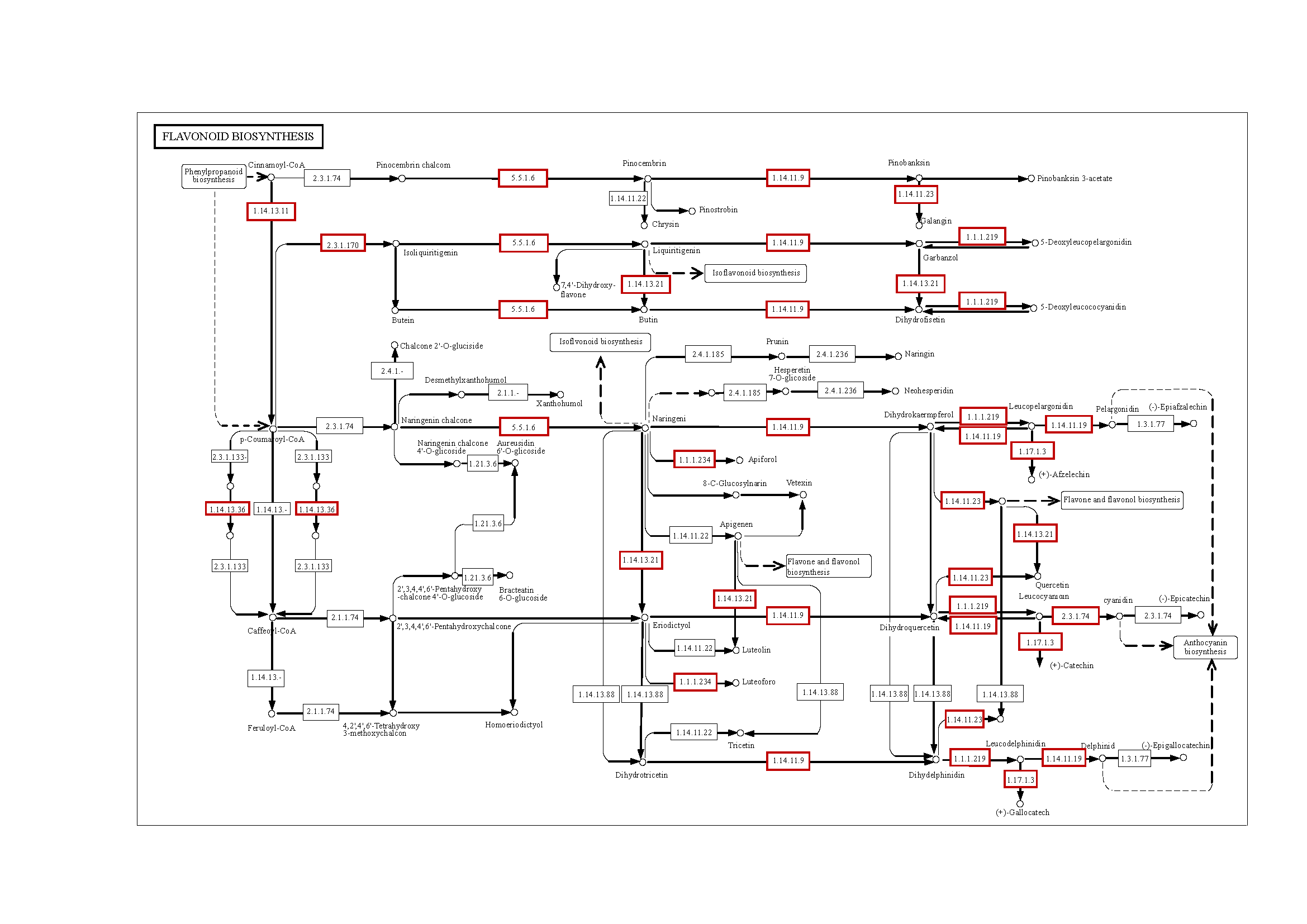


**Additional file Figure S12. Flavonoid biosynthesis KEGG pathways.** Each box shows enzymes involved in the pathway. Genes highlighted in red were detected from the 2,107 DEGs.

**
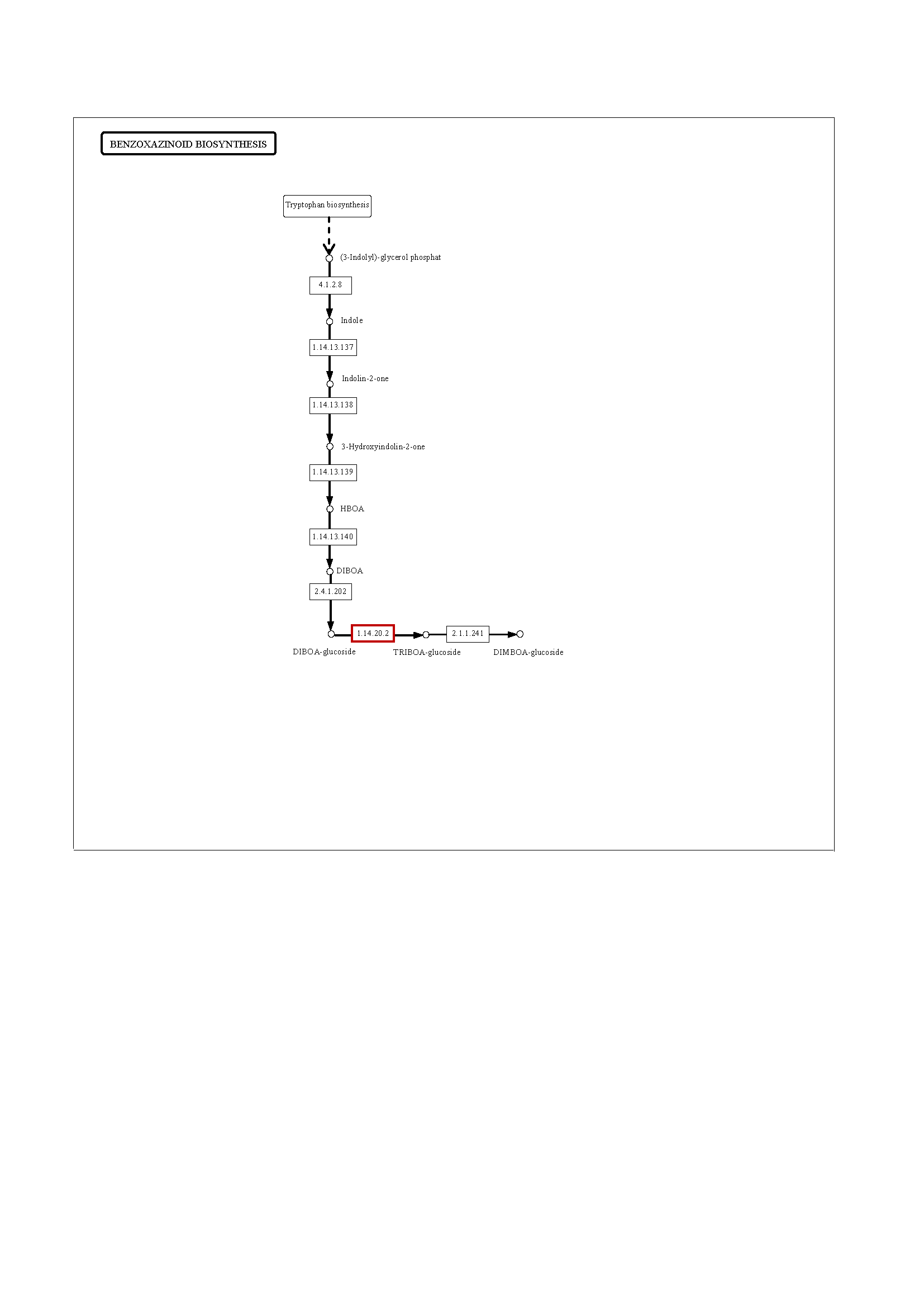
**

**Additional file Figure S13. Benzoxazinoid biosynthesis pathway.** Each box shows enzymes involved in the pathway. Genes highlighted in red were detected from the 2,107 DEGs.


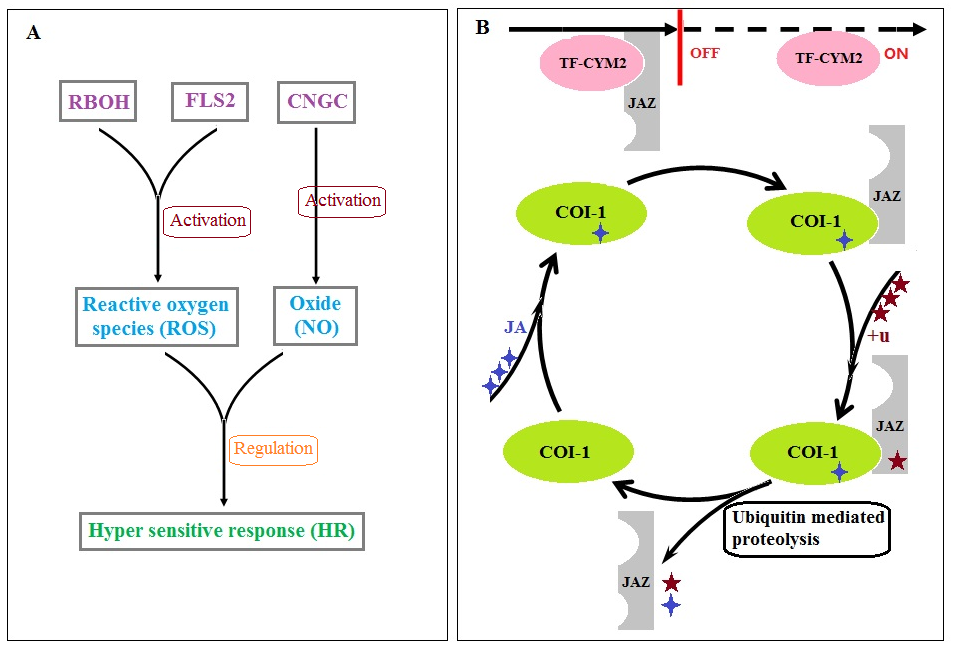


**Additional file Figure S14. Two key signaling pathways of Sunflowers response to *V. dahliae* infection. A:** The regulated network of Hyper sensitive response (HR) interact to reactive oxygen species (ROS) and oxide (NO) signaling pathway. **B:** COI1 is a key component of jasmonic acid- mediated signal transduction pathway leading to the dissociation between JAZ and transcription factor MYC2.
