# Supplementary material for: Large-scale transcriptome comparison of sunflower genes responsive to Verticillium dahliae
Source: BMC Genomics. 2017 Jan 6;18:42. doi: 10.1186/s12864-016-3386-7 (PMC5219742; doi:10.1186/s12864-016-3386-7)
Supplement: Additional file 2: Table S1. — Statistics of the reads in resistant genotype (S18) and susceptible genotype (P77). (DOCX 16 kb) [file 12864_2016_3386_MOESM2_ESM.docx]

**Additional table S1.** Statistics of the reads in resistant genotype (S18) and susceptible genotype (P77) at the different time points.

| **Sample** | **Total Reads** | **Total BasePairs** | **Total Mapped Reads (>96%)** | **Unique Match**  **(>68%)** | **Multi-position Match** | **Total Unmapped Reads** |
| --- | --- | --- | --- | --- | --- | --- |
| P10d | 17,246,655 | 2,470,961,960 | 16,631,633 | 11,829,088(68.59%) | 4,802,545(27.85%) | 615,022(3.57%) |
| P12h | 18,072,606 | 2,405,832,317 | 17,559,949 | 12,717,445(70.37%) | 4,842,504(26.79%) | 512,657(2.84%) |
| P24h | 15,122,736 | 1,962,278,477 | 14,673,221 | 10,511,027(69.50%) | 4,162,194(27.52%) | 449,515(2.97%) |
| P2d | 20,420,812 | 2,741,438,912 | 19,723,497 | 14,097,329(69.03%) | 5,626,168(27.55%) | 697,315(3.41%) |
| P3d | 15,966,081 | 2,170,830,783 | 15,433,196 | 11,045,062(69.18%) | 4,388,134(27.48%) | 532,885(3.34%) |
| P5d | 17,291,134 | 2,246,884,622 | 16,790,991 | 11,932,512(69.01%) | 4,858,479(28.10%) | 500,143(2.89%) |
| P6h | 14,615,138 | 1,985,149,822 | 14,214,366 | 10,194,701(69.75%) | 4,019,665(27.50%) | 400,772(2.74%) |
| PCK | 18,256,509 | 2,471,100,030 | 17,686,530 | 12,724,663(69.70%) | 4,961,867(27.18%) | 569,979(3.12%) |
| **Total**  **( P77 )** | **254766851** | **29922192643** | **246444874** | **176179302** | **70265572** | **8321977** |
| S10d | 16,777,526 | 2,242,634,942 | 16,196,233 | 11,442,008(68.20%) | 4,754,225(28.34%) | 581,293(3.46%) |
| S12h | 12,633,796 | 1,650,039,817 | 12,210,790 | 8,746,799(69.23%) | 3,463,991(27.42%) | 423,006(3.35%) |
| S24h | 12,499,849 | 1,674,759,973 | 12,118,037 | 8,738,437(69.91%) | 3,379,600(27.04%) | 381,812(3.05%) |
| S2d | 13,089,728 | 1,740,672,083 | 12,614,252 | 8,917,724(68.13%) | 3,696,528(28.24%) | 475,476(3.63%) |
| S3d | 16,696,486 | 2,200,864,994 | 16,116,081 | 11,433,917(68.48%) | 4,682,164(28.04%) | 580,405(3.48%) |
| S5d | 18,665,276 | 2,494,775,725 | 18,003,923 | 12,812,299(68.64%) | 5,191,624(27.81%) | 661,353(3.54%) |
| S6h | 13,691,990 | 1,869,800,503 | 13,221,819 | 9,461,213(69.10%) | 3,760,606(27.47%) | 470,171(3.43%) |
| SCK | 13,720,529 | 1,810,511,154) | 13,250,356 | 9,575,078(69.79%) | 3,675,278(26.79%) | 470,173(3.43%) |
| **Total**  **( S18 )** | **254766851** | **29922192643** | **246444874** | **176179302** | **70265572** | **8321977** |
