# Supplementary material for: Large-scale transcriptome comparison of sunflower genes responsive to Verticillium dahliae
Source: BMC Genomics. 2017 Jan 6;18:42. doi: 10.1186/s12864-016-3386-7 (PMC5219742; doi:10.1186/s12864-016-3386-7)
Supplement: Additional file 4: Table S3. — KEGG pathways of SCK-VS-PCK. (DOCX 16 kb) [file 12864_2016_3386_MOESM4_ESM.docx]

**Additional table S3.** Sixteen important KEGG pathways of SCK-VS-PCK.

| **NO.** | **Pathway** | **DEGs with pathway**  **annotation** | **All genes with pathway annotation** | **Pvalue** | **Qvalue** | **Pathway ID** |
| --- | --- | --- | --- | --- | --- | --- |
| 1 | Plant hormone signal transduction | 155 (6.71%) | 2157 (4.62%) | 2.157832e-06 | 0.0001327862 | ko04075 |
| 2 | Plant-pathogen interaction | 179 (7.75%) | 2566 (5.5%) | 2.213103e-06 | 0.0001327862 | ko04626 |
| 3 | Flavonoid biosynthesis | 38 (1.65%) | 365 (0.78%) | 1.461221e-05 | 0.0005844884 | ko00941 |
| 4 | Glycosylphosphatidylinositol(GPI)-anchor biosynthesis | 24 (1.04%) | 195 (0.42%) | 3.884378e-05 | 0.0010590677 | ko00563 |
| 5 | Starch and sucrose metabolism | 93 (4.03%) | 1235 (2.65%) | 4.412782e-05 | 0.0010590677 | ko00500 |
| 6 | Pentose and glucuronate interconversions | 55 (2.38%) | 641 (1.37%) | 6.171803e-05 | 0.0012343606 | ko00040 |
| 7 | Zeatin biosynthesis | 35 (1.52%) | 351 (0.75%) | 7.50063e-05 | 0.0012858223 | ko00908 |
| 8 | Phenylpropanoid biosynthesis | 58 (2.51%) | 736 (1.58%) | 0.0003729697 | 0.0055945455 | ko00940 |
| 9 | Phenylalanine metabolism | 32 (1.39%) | 345 (0.74%) | 0.0005367956 | 0.0071572747 | ko00360 |
| 10 | Biosynthesis of secondary metabolites | 322 (13.95%) | 5587 (11.97%) | 0.001793271 | 0.0215192520 | ko01110 |
| 11 | ABC transporters | 45 (1.95%) | 578 (1.24%) | 0.002000103 | 0.0218193055 | ko02010 |
| 12 | Vitamin B6 metabolism | 7 (0.3%) | 44 (0.09%) | 0.005508486 | 0.0535461231 | ko00750 |
| 13 | Other glycan degradation | 20 (0.87%) | 217 (0.46%) | 0.00580083 | 0.0535461231 | ko00511 |
| 14 | Stilbenoid, diarylheptanoid and gingerol biosynthesis | 32 (1.39%) | 406 (0.87%) | 0.006855389 | 0.0573749920 | ko00945 |
| 15 | Benzoxazinoid biosynthesis | 10 (0.43%) | 82 (0.18%) | 0.007171874 | 0.0573749920 | ko00402 |
| 16 | Ribosome biogenesis in eukaryotes | 62 (2.69%) | 916 (1.96%) | 0.008387566 | 0.0629067450 | ko03008 |
