# Supplementary material for: Large-scale transcriptome comparison of sunflower genes responsive to Verticillium dahliae
Source: BMC Genomics. 2017 Jan 6;18:42. doi: 10.1186/s12864-016-3386-7 (PMC5219742; doi:10.1186/s12864-016-3386-7)
Supplement: Additional file 5: Table S4-S6. — The summary of pairwise comparisons. (DOCX 17 kb) [file 12864_2016_3386_MOESM5_ESM.docx]

**Additional table S4.** The pairwise comparisons between inoculated and control samples for resistant genotype (S18) and susceptible genotype (P77), and pairwise comparisons between inoculated samples from resistant genotype (S18) and susceptible genotype (P77).

| **Comparision** | **Resulted Data set** | **Comparision** | **Resulted Data set** | **Comparision** | **Resulted Data set** |
| --- | --- | --- | --- | --- | --- |
| S6h-VS-SCK | RD6h | P6h-VS-PCK | SD6h | P6h-VS-S6h | D6h |
| S12h-VS-SCK | RD12h | P12h-VS-PCK | SD12h | P12h-VS-S12h | D12h |
| S24h-VS-SCK | RD24h | P24h-VS-PCK | SD24h | P24h-VS-S24h | D24h |
| S2d-VS-SCK | RD2d | P2d-VS-PCK | SD2d | P2d-VS-S2d | D2d |
| S3d-VS-SCK | RD3d | P3d-VS-PCK | SD3d | P3d-VS-S3d | D3d |
| S5d-VS-SCK | RD5d | P5d-VS-PCK | SD5d | P5d-VS-S5d | D5d |
| S10d-VS-SCK | RD10d | P10d-VS-PCK | SD10d | P10d-VS-S10d | D10d |

**Additional table S5.** The dynamic changes number of differentially expressed genes (DEGs) at series time points

| **Data set** | **Total number** | **Data set** | **Total number** | **Data set** | **Total number** |
| --- | --- | --- | --- | --- | --- |
| RD6h | 2885 | SD6h | 4663 | D6h | 8542 |
| RD12 | 2772 | SD12 | 1758 | D12 | 7820 |
| RD24 | 2247 | SD24 | 3421 | D24 | 5896 |
| RD2d | 5702 | SD2d | 5432 | D2d | 4995 |
| RD3d | 4576 | SD3d | 2158 | D3d | 5474 |
| RD5d | 3754 | SD5d | 3087 | D5d | 4957 |
| RD10d | 7734 | SD10d | 4830 | D10d | 6161 |

**Additional table S6.** The dynamic changes of number of up or down-regulated differentially expressed genes (DEGs) at series time points

| **Data set** | **Up** | **Down** | **Data set** | **Up** | **Down** | **Data set** | **Up** | **Down** |
| --- | --- | --- | --- | --- | --- | --- | --- | --- |
| RD6h | 1322 | 1563 | SD6h | 2948 | 1715 | D6h | 5469 | 3073 |
| RD12 | 1432 | 1340 | SD12 | 1160 | 598 | D12 | 5579 | 2241 |
| RD24 | 1397 | 850 | SD24 | 1462 | 1959 | D24 | 2229 | 3667 |
| RD2d | 2140 | 3562 | SD2d | 2444 | 2988 | D2d | 2789 | 2206 |
| RD3d | 2045 | 2531 | SD3d | 843 | 1315 | D3d | 2627 | 2847 |
| RD5d | 1847 | 1907 | SD5d | 1319 | 1768 | D5d | 2230 | 2727 |
| RD10d | 3277 | 4457 | SD10d | 1686 | 3144 | D10d | 2897 | 3264 |

S, S18; P, P77; Ck, control; Up, the numbers of DEGs up-regulated in inoculated samples; down, the numbers of DEGs up-regulated in inoculated samples.
